# The Treatment of Tuberculosis by Guaiacol

**Published:** 1892-04

**Authors:** 


### THE TREATMENT OF TUBERCULOSIS BY GUAIACOL.

We have received from Professor Max Schueller, of Berlin, a report embodying the results of his treatment of tuberculosis with guaiacol.

Guaiacol is the principal constituent of creasote, a clear liquid, easily soluble, with an unpleasant taste and odor. In pulmonary tuberculosis he gives children two to three drops, adults three to five drops of pure guaiacol four times daily in salt water, milk, bouillon or wine. It is not advisable to give it in pill form, or in capsules. Patients should take occasionally with the guaiacol expectorants, digitalis, and antipyretics, when these seem to be indicated. After some months' treatment there can usually be noted an increased, but easier, expectoration, a gradual change of the lumpy, purulent sputum into catarrhal mucus, clearing of resonance, relief of coughing, increased body weight, disappearance of bacilli, and general constitutional improvement. Marked improvement in all symptoms may be noted after four or five weeks' treatment, but the regular course of treatment should extend over six or eight months, and should even be continued after the disappearance of the tubercle bacilli and the local symptoms of the disease. He records eighteen cases in patients with pulmonary tuberculosis, some of whom have been under observation for several years, and who proved to be entirely well at repeated examinations.

In surgical tuberculosis he has tried the guaiacol treatment, and found a number of cases in which were improvement without resort to operation. In some cases he considers an operation either advisable or necessary in conjunction with the guaiacol. Large tuberculous glandular tumors, not yet caseated, may be made to disappear in three to six months.

Dr. Schueller has been conducting his experiments now for twelve or fourteen years, with the result, probably, of having inaugurated a line of treatment for tuberculosis which promises more than any other. It is not claimed for guaiacol that it is a specific, but it does appear to be an agent capable of exerting a favorable influence over many cases, and of establishing a cure in others when taken in their incipiency.

